# Factors associated with failure of hydrostatic reduction in children with ileocolic intussusception

**DOI:** 10.1186/s12887-026-07146-1

**Published:** 2026-06-16

**Authors:** Jie Xiong, Hui Yang, Tianliang Li, Gaolian Hu, Jun Wang, Hong Yang, Pinghui Zhou, Qian Tan, Xin Wang

**Affiliations:** 1 Department of Pediatric Surgery, Children’s Emergency Center, The First People’s Hospital of Zigong, Zigong, 643000 China; 2 Emergency and Cancer Treatment Center, The Third People’s Hospital of Zigong, Zigong, 643020 China; 3 Department of Medical Ultrasound, The First People’s Hospital of Zigong, Zigong, 643000 China; 4 Department of Medical Ultrasound, The Third People’s Hospital of Zigong, Zigong, 643020 China

**Keywords:** Pediatric, Ileocolic intussusception, Hydrostatic reduction, Risk factor

## Abstract

**Background/objectives:**

There remains controversy regarding the risk factors for failed hydrostatic enema reduction; therefore, we aimed to identify additional factors associated with unsuccessful hydrostatic enema reduction in children with ileocolic intussusception.

**Methods:**

This study was conducted retrospectively in two tertiary centers. Data were collected from patient charts or electronic medical records and consisted of pediatric intussusception cases treated with hydrostatic reduction during January 2021 and January 2025. Univariate and multivariate analyses, incorporating stepwise logistic regression, were conducted.

**Results:**

Two hundred thirty-one patients with ileocolic-type intussusception were included and treated by ultrasound-guided hydrostatic reduction at two different institutions. Hydrostatic reduction was successful in 199 patients (86.2%), failed in 32 (13.8%). All patients were successfully discharged with uneventful recoveries. On multivariate analysis, under 12-month-old(OR = 58.106,*P* < 0.001 95%CI,14.166-238.338),an Onset of symptoms>48 h (OR = 7.070,*P* = 0.014 95%CI,1.491–33.517), previous history of intussusception (OR = 42.721, *P* < 0.001 95%CI,5.729-318.572), constipation (OR = 31.488, *P* < 0.001 95%CI,5.597-177.137), and bowel Wall Thickening on US(OR = 8.177, *P* = 0.015 95%CI,1.513–43.553) were significantly associated with failed hydrostatic enema reduction.

**Conclusions:**

An age of under 1 year, previous history of intussusception, onset of symptoms, constipation, and bowel wall thickening on US were risk factors for failed hydrostatic reduction of ileocolic intussusception. Older children with long-term recurrent intussusception are at high risk of pathological leading points(PLPs) and hydrostatic reduction failure, requiring close pediatric surgical attention. Patients with these findings warrant early surgical consultation or transfer to a facility with pediatric surgical capabilities.

## Introduction

Intussusception is a common acute abdominal emergency in pediatric surgery that causes bowel obstruction in children, with an incidence of 56/100,000 patients [1]. Delayed diagnosis and treatment may cause bowel necrosis and even death [2]. Compared with surgery, enema reduction with a higher success rate and less trauma is the first-line clinical treatment for pediatric intussusception. Abdominal ultrasound is the first-line modality for rapid and accurate diagnosis of ileocolic intussusception in children; however, no more than 25% of affected patients exhibit the classic four clinical manifestations: abdominal pain, vomiting, bloody stool, and palpable abdominal mass. The lack of typical clinical signs is a leading cause of pre-hospital misdiagnosis and delayed intervention [3, 4]. Delayed treatment causes bowel wall edema and ischemia, increasing the difficulty of hydrostatic enema reduction, even leading to bowel necrosis and perforation [5]. Existing literature shows that the success rate of hydrostatic enema reduction ranges from 12.15% to 96.7% [6, 7].

To date, numerous clinical studies and academic discussions have been conducted on the risk factors for failed hydrostatic reduction or pneumatic reduction in pediatric intussusception, with inconsistent conclusions [7–10]. Patient-related factors including age, onset of symptoms, bloody diarrhea, and constipation, remain controversial, while sonographic factors such as bowel wall thickness, trapped fluid in intussusception, and peritoneal fluid are still under discussion [7, 11]. With advancing enema reduction techniques and growing clinical experience, the choice of enema modalities for pediatric intussusception has shifted across medical centers. Over the past 8 years, our two centers have used hydrostatic enema reduction as first-line treatment because of its simplicity, safety, efficacy, and radiation-free nature, while other institutions prefer pneumatic enema reduction for pediatric intussusception [9, 11].

We attempted to identify variables related to the failure of hydrostatic reduction of ileocolic-type intussusception in pediatric patients. We collected data including demographic characteristics, clinical signs, and ultrasound features of enrolled children, and followed up their treatment process and therapeutic outcomes. These findings facilitate the early identification of pediatric patients at high risk of failed hydrostatic enema reduction, for whom early referral to a specialized tertiary pediatric hospital with dedicated pediatric surgical care is clinically warranted.

## Materials and methods

An analytical study with retrospective data was performed in two tertiary hospitals. Data was collected from patient charts or electronic medical records and consisted of pediatric intussusception cases treated with hydrostatic reduction during January 2021 and January 2025.Patients were excluded from this study if they had incomplete medical records or were not ileocolic-type intussusception.Data information was collected, including basic demographics, symptoms, ultrasound findings, operative outcomes, and etiology.

In our practice, ileocolic-type intussusception was diagnosed by using an ultrasound conducted by a senior sonographer following the clinical guidelines. And hydrostatic reduction was done as described by Xie et al. [7] under continuous ultrasound guidance. Procedures were conducted with the child in a fully awake state without sedation or general anesthesia.All hydrostatic reduction procedures in both two centers were performed according to the same standardized protocol.The protocol specified uniform parameters, including enema fluid temperature, infusion pressure, maximum procedure duration, and criteria for successful reduction.The success of reduction was determined by the disappearance of the target appearance and the visualization of the fluid reflux into the distal ileum from the cecum to the ileum through the ileocecal valve, and fluid filling of small bowel loops.Failed reduction was defined as a remaining intussusception mass after 3 separate attempts, then surgical management was pursued. Each hydrostatic reduction attempt lasted 5 min, with a mandatory 20-min interval between repeated attempts for failed reduction. According to hydrostatic reduction outcomes, all patients were classified into the successful or failed groups. Onset of symptoms was defined as time (in hours) from the first reported symptoms to hydrostatic reduction for intussusception.Recurrent intussusception was defined as recurrence occurring within 72 h or during the hospital stay after successful hydrostatic reduction.Previous history of intussusception was defined as recurrence of ileocolic-type intussusception after the last discharge from the hospital.Bowel wall thickening was defined as a bowel wall thickness greater than 7.2 mm on ultrasound, consistent with criteria reported in previous studies [12].

The study was approved by the ethical committee of the two institutions; the requirement for informed consent was waived owing to the retrospective nature. All methods were performed in accordance with the relevant guidelines and regulations.

SPSS version 26.0(IBM Corp., Armonk, NY, USA)was performed for all data analyses. The descriptive data was reported, with continuous variables reported as means ± SD and categorical variables presented as frequencies(percentages). An independent t-test was used to compare continuous variables between the two groups. Categorical variables were examined using either the chi-square test or Fisher’s exact test. Stepwise logistic regression was performed to identify independent risk factors. The *p* < 0.05 were set to be statistically significant.

## Results

During this study period, 231 patients(165 boys and 66 girls) with ileocolic-type intussusception were included and treated by ultrasound-guided hydrostatic reduction at two different institutions. Hydrostatic reduction was successful in 199 patients (86.2%), failed in 32 (13.8%). Among the patients, the mean body weight of the patients was 14.38 ± 4.34 kg(Successful Group) and 14.13 ± 4.37(Failed Group). 194(84)% of the included pediatric patients were older than 1 year of age, and 15(6.5%) patients had received treatment (≥ 48 h after the first onset of symptoms). The symptoms collected were bloody stools, constipation, and previous history of intussusception (39.8%,8.22% and 4.7%respectively). The majority of the patients were aged 0–12 months. Details of the other variables and univariate analysis are provided in Table 1.

**Table 1:** Univariate comparison of Successful versus Failed group (*n* = 231)

Variable	Successful Group, (*n* = 199)n (%)	Failed Group, (*n* = 32)n (%)	*p*-value
Sex			0.434
Man	144(72.4)	21(65.6)	
Female	55(27.6)	11(34.4)	
Age(months)			< 0.001*
< 12	15(7.5)	22(84)	
≥ 12	184(92.5)	10(16)	
Weight (Kg), mean$\:\pm\:$SD^a^	14.38$\:\pm\:$4.34	14.13$\:\pm\:$4.37	0.771
Onset of symptoms(hours)^b^			0.009*
< 48 h	190(95.5)	26(81.3)	
≥ 48 h	9(4.5)	6(18.7)	
Bloody stools			0.380
Yes	77(38.7)	15(46.9)	
No	122(61.3)	17(53.1)	
Constipation^b^			< 0.001*
Yes	9(4.5)	10(31.2)	
No	190(95.5)	22(68.8)	
Previous history of intussusception^b^			< 0.001*
Yes	3(1.5)	8(25)	
No	196(98.5)	24(75)	
Peritoneal fluid on US			0.009*
Yes	35(17.6)	12(37.5)	
No	164(82.4)	20(62.5)	
Trapped fluid in intussusception on US^b^			0.015*
Yes	18(9)	8(25)	
No	181(91)	24(75)	
Bowel Wall Thickening on US^b^			0.024*
Yes	12(6)	6(18.7)	
No	187(94)	26(81.3)	

*US* ultrasound

* denotes statistical significance

^a^ independent t-test for continuous variables

^b^ fisher exact test for <5 value in contingency table

The success rate of hydrostatic reduction in intussusception treated was low in < 12month-old patients. Univariate analyses revealed a significant increase in constipation(*P* < 0.001) in the failed group.No significant differences in the outcomes were observed by sex (*p* = 0.434), body weight (*p* = 0.771), or presence of bloody stools(*p* = 0.380). The onset of symptoms (*P* = 0.009) and previous history of intussusception (*p* < 0.001) were significantly correlated with the hydrostatic reduction outcomes. On ultrasound imaging, free intraperitoneal fluid, fluid trapped within the intussusception, and intussusceptum bowel wall thickening were identified as findings predictive of failed reduction.

In order to assess for independent factors of failed reduction, a stepwise logistic regression analysis was performed in Table 2.we found that the significant independent factors for failure of hydrostatic reduction of intussusception were under 12-month-old (OR = 58.106,*P* < 0.001),an Onset of symptoms>48 h (OR = 7.070,*P* = 0.014), previous history of intussusception (OR = 42.721, *P* < 0.001), constipation (OR = 31.488, *P* < 0.001), and bowel Wall Thickening on US(OR = 8.177, *P* = 0.015).

**Table 2:** Stepwise logistic regression model for significant predictors of failing reduction

Variable	OR^a^	95%CI^b^	*p*-value
Age < 12 months	58.106	14.166−238.338	< 0.001
Previous history of intussusception	42.721	5.729−318.572	< 0.001
Onset of symptoms	7.070	1.491–33.517	0.014
Constipation	31.488	5.597−177.137	< 0.001
Bowel Wall Thickening on US^c^	8.177	1.513–43.553	0.015

^a^ odds ratio

^b^ confidence interval

^c^ ultrasound

In our series, the treatments and findings for all patients with failed reduction or successful reduction were shown in Table 3.Of the 199 successful reduction patients,189(94.97%)had reduction with a single attempt, 7༈3.52%༉with two attempts, and 3༈1.51%༉with three attempts.2.16% (5/231) developed recurrent intussusception within 72 h or during the hospital stay after successful hydrostatic reduction. Among these, 2 experienced a single episode of recurrence and 1 experienced two episodes of recurrence in the successful group. In the failed reduction group, both of the 2 patients developed two recurrences.For patients in the failed reduction group, 12 underwent intestinal resection, while 20 received manual reduction during surgical intervention. Of the 12 pediatric patients who underwent intestinal resection, the underlying surgical indications were intestinal necrosis in 3 cases, intestinal polyps in 4 cases, Meckel’s diverticulum in 3 cases, intestinal duplication cyst in 1 case, and intestinal lymphoma in 1 case. All 231 patients were successfully discharged with uneventful recoveries.

**Table 3:** Outcomes of all treated patients in Successful and Failed Groups

Characteristic	*n* (%)
Successful hydrostatic reduction (*n* = 199)	
Single attempt reduction	189(94.97%)
Two attempts reduction	7(3.52%)
Three attempts reduction	3(1.51%)
Failed hydrostatic reduction (*n* = 32)	
Surgical intervention	32
- Intestinal resection	12
- Manual reduction	20
Recurrent intussusception(*n* = 5)	
single episode of recurrence	2
two episodes of recurrence	3
PLPs identified in failed group (*n* = 9)	
Intestinal polyp	4
Meckel’s diverticulum	3
Duplication cyst	1
Lymphoma	1

In our study, history of intussusception was a significant independent factor of an unsuccessful reduction.The details of the history of intussusception are presented in Table 4.Of the 231 reduction patients,11(4.76%)had a positive prior history of intussusception. Among pediatric patients with a prior history of intussusception who experienced disease recurrence, 27.27% (3/11) of recurrences occurred within 1 month of the initial episode, 54.54%(6/11) occurred between 1 and 3 months, and the remaining 18.18%(2/11) occurred more than 3 months after the initial onset.

**Table 4:** Analysis of pediatric patients with a positive prior history of intussusception

Time to recurrence	Successful group(*n* = 3)	Failed group(*n* = 8)
< 1 month	2	1
1–3 month	1	5
> 3month	0	2

## Discussion

Identifying independent risk factors for failed reduction is essential to reduce treatment delays and improve salvage outcomes in patients with Ileocolic intussusception. Multiple studies have assessed potential predictors of failed hydrostatic reduction in children with intussusception, with widely variable results. Age has been identified as an independent risk factor, yet the stratification criteria and cut-off values reported across these studies remain inconsistent. In the study by Kusmayadi et al. [8], the authors reported an association between age and small bowel diameter, with smaller diameter observed in younger children. A smaller bowel diameter may theoretically increase the risk of failure during hydrostatic reduction. However, their conclusion that children aged < 3 years had a higher success rate of hydrostatic reduction may be biased by their small sample size—only 9 patients with successful reduction—and an overall low success rate of only 16.1%. Salahoudine et al. [13] linked age > 2 years to an increased surgical rate, while others study found that greater failure of hydrostatic reductions occurred in those under one year of age [14, 15]. Our results confirm that children aged < 12 months have an increased risk of failed reduction. In addition to the narrower small intestinal lumen in young infants predisposing to hydrostatic reduction failure, we hypothesize that an equally important factor is the overly conservative approach frequently employed by non-specialized pediatric centers or less experienced operators during the procedure.

Our study confirms that a history of intussusception is an important independent risk factor for failed hydrostatic reduction in children with intussusception.Gadgade et al. [10] included 158 pediatric patients, 2.5% of whom had a positive prior intussusception history, with no statistically significant effect of this factor reported in their analysis. However, among the 4 children with a previous history of intussusception, 3 experienced failed hydrostatic reduction and subsequently underwent surgical treatment. The lack of statistical significance in their study is likely attributable to the limited overall sample size. Despite the relatively small sample size of patients with a positive intussusception history, our results remain clinically relevant. In this cohort, 27.27% of intussusception recurrences occurred within 1 month, 54.54% between 1 and 3 months, and 18.18% beyond 3 months. Our analysis showed that 7 out of 8 patients with both failed reduction and a positive prior history had confirmed pathological lead points (PLPs), all of whom were aged over 5 years. This highlights the need for pediatric surgeons to maintain high vigilance for underlying PLPs in older children with a history of intussusception, which may be driven by the progression of previously unrecognized PLPs.

In our multivariable analysis, symptom duration > 24 h was an independent risk factor for failed hydrostatic reduction, which diverges from previous reports.In a Hong Kong-based patient cohort, Wong et al. [3] found that a mean symptom duration of 2.3 days did not impact the success rate of hydrostatic enema reduction. In the study by Zhang et al. [16], symptom duration longer than 24 h was statistically significant in univariable analysis, but showed no statistical significance in multivariable analysis. Nevertheless, the authors still recommended that a more conservative strategy for hydrostatic enema reduction should be adopted in pediatric patients with symptom duration exceeding 24 h. Differences in the definition of symptom duration contribute to divergent findings across studies.Lampl et al. [17] defined the delay to attempted reduction as the time interval from the patient’s initial presentation to the emergency department, a definition distinct from that employed in our study.In addition, we posit that the non-specific and delayed clinical presentation of intussusception, masking of symptoms by concomitant gastrointestinal diseases (e.g., acute gastroenteritis), and underestimation of actual symptom duration due to caregiver oversight are also important contributors to between-study heterogeneity in risk factor analyses, as well as systematic bias in symptom duration assessment in clinical practice.

Previous studies have investigated the association between patient-related factors (including abdominal pain, vomiting, fever, bloody diarrhea, abdominal mass, constipation, and dehydration) and the failure of hydrostatic reduction, with conflicting conclusions [10, 18–20]. Our study demonstrated that constipation is an independent risk factor for hydrostatic reduction failure, in line with the previously published results from Fallon and colleagues [11]. The hardened stool absorbs a portion of the enema fluid, causing instability of the hydrostatic pressure during enema reduction. Meanwhile, constipation-related relaxation of the anal sphincter leads to fluid leakage throughout the enema process, resulting in inadequate intraluminal pressure and ultimately failed reduction. Furthermore, the increased intraluminal solid contents can attenuate the transmission of the applied hydrostatic pressure.Therefore, prior to the performance of hydrostatic enema reduction, a single optimized pre-procedure cleansing enema for the evacuation of retained hardened stool is recommended to enhance the success rate of the reduction procedure.

The abdominal ultrasound findings most commonly described in the literature for cases of intussusception were included in this study. In the univariate logistic regression analysis of our cohort, the presence of peritoneal fluid, trapped fluid within the intussusception, and bowel wall thickening all exhibited statistical significance. However, in the subsequent multivariable logistic regression analysis, only bowel wall thickening was identified as an independent statistically significant predictor of failed hydrostatic enema reduction. This result was inconsistent with the previously reported [8, 21].The reason may be that the predictive factors are related to the bowel wall thickening.Bowel wall thickness exceeding 7.2 mm indicates increased bowel wall stiffness and decreased intestinal compliance, which may contribute to failed hydrostatic enema reduction. However, bowel wall stiffness can be quantitatively measured with shear-wave elastography (SWE), and this technique has been shown to predict the success rate of hydrostatic enema reduction [22].

With the advancement of enema reduction techniques and improved success rates, enema reduction has become the first-line treatment for pediatric intussusception [23]. Hydrostatic enema reduction is widely used for its simplicity, safety, efficacy, and radiation-free nature [24, 25]. The success rate of hydrostatic enema reduction in our study was 86.2%, consistent with previously published studies [16, 26]. Ultrasound-guided hydrostatic enema reduction can be safely attempted repeatedly, with no restrictions on procedural duration [25]. Among the 199 pediatric patients in the successful reduction group, 189 (94.97%) achieved successful hydrostatic enema reduction after a single attempt, 7 (3.52%) after 2 attempts, and 3 (1.51%) after 3 attempts. The overall success rate of repeated hydrostatic enema reduction attempts after initial failure was 23.8%, indicating that repeated enema reduction attempts can provide clinical benefit for children with intussusception. Children with prior failed hydrostatic enema reduction for intussusception may benefit from referral to a dedicated children’s hospital for a repeat reduction attempt by an experienced clinician with pediatric surgical backup on standby.

PLPs play a critical role in the failure of hydrostatic enema reduction [1].In the study by Blakelock and Beasley [27], 60% of PLPs were found to occur in children aged 5 to 14 years. Underlying PLPs are often associated with atypical clinical manifestations of intussusception, and the incidence of PLP-induced intussusception increases with age, particularly in children older than 1 year. In our series, 9 had identifiable PLPs, including intestinal polyps in 4 cases, Meckel’s diverticulum in 3 cases, intestinal duplication cyst in 1 case, and intestinal lymphoma in 1 case. Of these 9 patients, 7 had a prior history of intussusception, and all were older than 5 years of age, supporting the notion that PLPs may be present in older children with long-term recurrent intussusception.

There are several limitations to our study. First, all data were collected retrospectively, making associated bias unavoidable. Second, the overall sample size of our study was relatively small, which is attributable to the relative rarity of pediatric intussusception in clinical practice. Finally, laboratory markers (WBC, ESR, CRP, etc.) were not included in the analysis, and the exclusion of these factors may have influenced the final results. Despite these limitations, our study improves the understanding of risk factors for failed hydrostatic reduction in pediatric patients. And based on our results, the development of evidence-based guidelines at the institutional level appears warranted.

## Conclusions

Our study found that an age of under 1-year-old, previous history of intussusception, onset of symptoms, constipation, bowel wall thickening on US were risk factors for failure of hydrostatic reduction of ileocolic-type intussusception.Pediatric surgeons should pay close attention to older children with long-term recurrent intussusception, who are at high risk of PLPs and failed hydrostatic enema reduction.Clinicians in community settings or hospitals without adequate pediatric surgical capabilities should prioritize early transfer to higher-level care when relevant resources are accessible.

## Data Availability

The data presented in this study are available on request from the corresponding author. The data are not publicly available due to ethical restrictions.

## Abbreviations

US: Ultrasound
HRA: Hydrostatic reduction attempt
PLPs: Pathological lead points
WBC: White Blood Cell
ESR: Erythrocyte sedimentation rate
CRP: C-reactive protein
